# Medical education trends for future physicians in the era of advanced technology and artificial intelligence: an integrative review

**DOI:** 10.1186/s12909-019-1891-5

**Published:** 2019-12-11

**Authors:** Eui-Ryoung Han, Sanghee Yeo, Min-Jeong Kim, Young-Hee Lee, Kwi-Hwa Park, Hyerin Roh

**Affiliations:** 1 Department of Medical Education, Chonnam National University Medical School, 264 Seoyang-ro, Hwasun-eup, Hwasun-gun, Jeollanam-do 58128 South Korea; 2 Department of Medical Education, School of Medicine, Kyungpook National University, 680 Gukchaebosang-ro, Jung-gu, Daegu, 41944 South Korea; 3 Department of Medical Education and Neurology, Kosin University College of Medicine, 262, Gamcheon-ro, Seo-gu, Busan, 49267 South Korea; 4 Medical Education Center, College of Medicine, Korea University, 73 Goryeodae-ro, Seongbuk-gu, Seoul, 02841 South Korea; 5 Department of Medical Education, Gachon University College of Medicine, 38 Dokjeom-ro 3beon-gil, Namdong-gu, Incheon, 21565 South Korea; 6 Department of Medical Education and the Institute for Medical Humanities, Inje University College of Medicine, 75-Bokji-ro, Busanjin-gu, Busan, 47392 South Korea

**Keywords:** Undergraduate medical education, Technology, Humanities, Integration, Societies, Self-directed learning

## Abstract

**Background:**

Medical education must adapt to different health care contexts, including digitalized health care systems and a digital generation of students in a hyper-connected world. The aims of this study are to identify and synthesize the values that medical educators need to implement in the curricula and to introduce representative educational programs.

**Methods:**

An integrative review was conducted to combine data from various research designs. We searched for articles on PubMed, Scopus, Web of Science, and EBSCO ERIC between 2011 and 2017. Key search terms were “undergraduate medical education,” “future,” “twenty-first century,” “millennium,” “curriculum,” “teaching,” “learning,” and “assessment.” We screened and extracted them according to inclusion and exclusion criteria from titles and abstracts. All authors read the full texts and discussed them to reach a consensus about the themes and subthemes. Data appraisal was performed using a modified Hawker ‘s evaluation form.

**Results:**

Among the 7616 abstracts initially identified, 28 full-text articles were selected to reflect medical education trends and suggest suitable educational programs. The integrative themes and subthemes of future medical education are as follows: 1) a humanistic approach to patient safety that involves encouraging humanistic doctors and facilitating collaboration; 2) early experience and longitudinal integration by early exposure to patient-oriented integration and longitudinal integrated clerkships; 3) going beyond hospitals toward society by responding to changing community needs and showing respect for diversity; and 4) student-driven learning with advanced technology through active learning with individualization, social interaction, and resource accessibility.

**Conclusions:**

This review integrated the trends in undergraduate medical education in readiness for the anticipated changes in medical environments. The detailed programs introduced in this study could be useful for medical educators in the development of curricula. Further research is required to integrate the educational trends into graduate and continuing medical education, and to investigate the status or effects of innovative educational programs in each medical school or environment.

## Background

Medical education must evolve because future physicians will encounter patients in quite different health care contexts from the present. Ubiquitous and digitalized health care systems allow both physicians and patients to access biomedical information easily [1]. Exponentially expanding medical knowledge requires physicians not to recall, but to update, what they know and select the right information from a surplus of options. Artificial intelligence will reduce the efforts required by physicians to interpret digital data and improve their ability to establish a diagnosis and prognosis. Therefore, the non-analytical, humanistic aspect of medicine will come to be more emphasized because it is hard to replace it with technology [2, 3]. Moreover, advanced medical technology leads to physicians encountering a growing number of elderly people and latent patients with chronic conditions and comorbidities due to their prolonged life span [4]. Globalization has led to physicians facing unfamiliar disease profiles or contexts that were not common in regional communities [5, 6]. Future medical education should be restructured to align with such inexorable changes by considering learners who will be working in digitalized health care systems.

In a digital world, learners are quite different from previous generations. They are digital learners who have grown up with and are hyper-connected through the Internet [1, 7–9]. Although they may spend a lot of time playing computer games alone, they still regard social interaction as very important so much that they prefer working in groups and sharing the details of their activities with others in an online community as well as in the classroom [8–10]. They tend to prefer feedback on their achievements and express a need for individual psychosocial support [10]. Educators should respond to the changing nature of learners by using more team-based, collaborative, and game-based learning rather than insisting on only traditional teaching methods [7]. Therefore, educators need to identify and employ suitable teaching strategies to engage and keep the attention of these students.

There have been several reports and studies on future medical education [11–13]. The Institute of Medicine organized a multidisciplinary summit focused on integrating a core set of competencies—patient-centered care, interdisciplinary teams, evidence-based practice, quality improvement, and informatics—into education for health professionals and recommended a mix of approaches related to the oversight processes, the training environment, research, public reporting, and leadership [11]. “Training Tomorrow’s Doctors” demonstrated the challenges facing the educational mission and recommended principles for academic health centers, accrediting organizations and similar groups, and public policy [12]. In 2010, the Carnegie Foundation addressed the related challenges and made recommendations on achieving excellence in medical education, which traced four themes in Flexner’s work: standardization and individualization, integration, habit of inquiry and improvement, and identity formation [13]. However, these reports abstractly presented the goals and directions of future medical education. Researchers have rarely conducted practical investigations into what global medical educators are conceiving and implementing in order to prepare for the future of advanced technology and expanding knowledge. Furthermore, there have been few reports on how to educate the next generation in a way that fits their characteristics [1, 8–10].

According to an open-systems perspective on the social system theory, schools are open systems that are influenced by environments and depend on exchanges with the environment to survive [14]. We hypothesized that medical schools would use resources from the environment, such as students, teachers, and instructional materials in the changing world. The students would be transformed by the school system, including the teaching and learning as shaped by social and environmental forces, into educated graduates who then contribute to the broader environment.

An integrated review of the current educational activities could help educators and policy makers to grasp the chief educational trends in preparation for the future because it could contribute to the presentation of varied perspectives on changing medical education. Therefore, we investigated various innovative programs or courses and identified and synthesized the values they presented. We will use the knowledge gained to suggest representative educational programs. The specific research questions were as follows:

1. What are the trends in medical education to foster future physicians in the era of advanced technology and expanding knowledge?
2. Which programs have been implemented specifically in line with each future trend?

## Methods

We chose integrative review as the methodology to identify and synthesize future medical education trends from different types of research. Integrative review, developed by Whittemore et al. [15], is an unique approach to combining data from various research designs including experimental and non-experimental research [15, 16]. This method proceeds through the stages of problem identification, literature search, data evaluation, data analysis, and presentation [15]. The study was exempt from review by the Chonnam National University Hospital Institutional Review Board (IRB No. CNUH–EXP–2018–042).

### Literature search

A search strategy was designed with input from six authors. ERH and SY searched for articles on PubMed, Scopus, Web of Science, and EBSCO ERIC. The key search terms were “(undergraduate medical education OR medical education) AND (future OR 21^st^ century OR millennium) AND (curriculum OR teaching OR learning OR assessment).” We collected articles published between 2011 and 2017 because there was a twofold increase in electronic health records and an explosion in digital information generated in 2011 [17, 18]. We downloaded article lists and then completed the search in the software program Excel in order to facilitate the review of the data. Besides various computerized databases, journal hand searches, citation-index searches, and Internet searches were used to conduct more comprehensive searches [19].

#### Inclusion and exclusion criteria

Both experimental and non-experimental studies in English were included. The research articles had to describe curricula or the methods of teaching, learning, and assessment in undergraduate medical education. Among them, we included research results on new teaching methods as well as future-oriented teaching methods. We excluded teaching methods such as problem-based learning (PBL) and evidence-based learning, which have been used for many years at medical schools. However, if the PBL method attempted to combine new technologies, it could be included. On the other hand, even if information technology was used, we excluded research if it did not involve new educational strategies, new ideas, or meaningful attempts to improve future education.

We excluded review articles, dissertations, letters, opinions or perspectives, and commentaries. Articles were eliminated if their subjects were restricted to nursing, dentistry, or other specialized health profession students, or if they studied only graduate or continuous medical education. Additionally, we excluded articles that focused on the selection of students and their well-being or career choices.

#### Data selection

After duplicates were removed, ERH and SY screened titles and abstracts based on the inclusion and exclusion criteria individually and in full-text form when it was necessary to confirm the exact content. These articles were coded according to the relevant criteria for this integrative review (three-point scale: high, low, or indefinite) [15]. We met and held a discussion to select the articles that presented educational programs or developed a new and innovative program to cultivate future physicians who would work in an era of rapid technological change. Articles that suggested innovative values for medical educators to implement in the curricula were also included. In cases of disagreement, another researcher (HR) was consulted.

#### Search outcomes

**Fig. 1:**
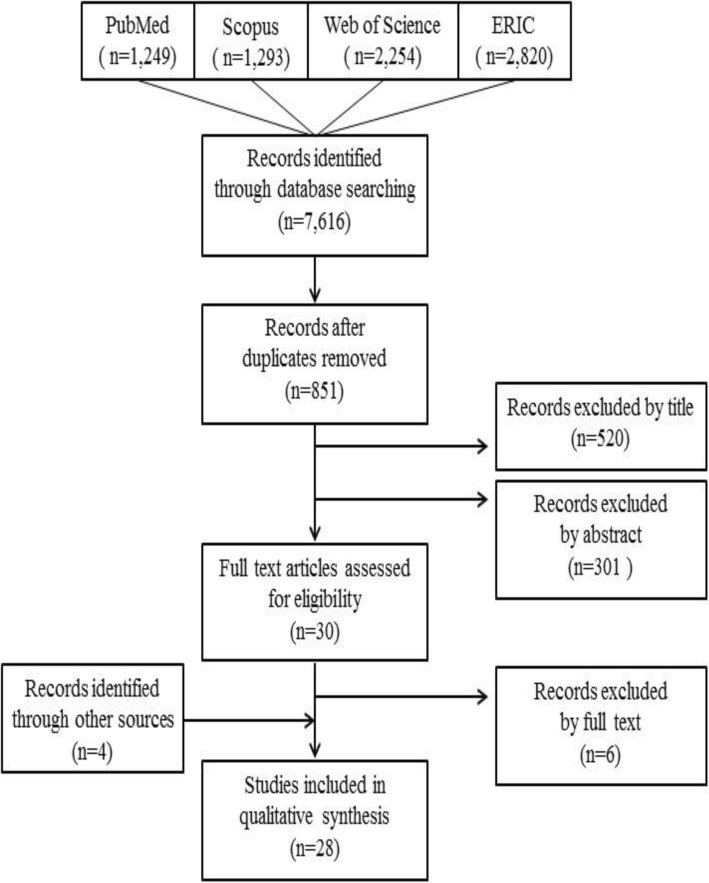
Flowchart of literature search

A total of 7616 articles were initially identified from the electronic databases. A total of 851 articles remained after removing duplicates. ERH and SY screened them independently according to the inclusion and exclusion criteria and extracted 30 articles from titles and abstracts. ERH, SY, and HR read the 30 full-text articles and discussed the representative educational programs. Thereafter, we removed six articles and added four to replace them through other sources including journal hand searches, citation-index searches, and Internet searches. Finally, 28 articles remained for full analysis. The result of search strategy is shown in Fig. 1.

### Data evaluation

A modified Hawker’s evaluation form was used to evaluate the quality of the data [20]. This evaluation form consists of nine questions. In our study, eight items were used; the “ethics and bias” question was removed since the topic of our paper was not health care-related issues but non-experimental educational curriculum trends. The questions address abstracts and titles, introductions and aims, methods and data, sampling, data analysis, results, transferability and generalizability, and implications and usefulness. Ratings were evaluated as 4 = good, 3 = fair, 2 = poor, and 1 = very poor. The overall methodological quality was determined based on the average score over the eight items (4.00–3.51 = good; 3.50–2.51 = fair; 2.50–1.51 = poor; and 1.50–1.00 = very poor). ERH and SY independently evaluated each article using the modified Hawker’s evaluation form and completed an unbiased data extraction table. Articles rated as less rigorous were not excluded as this study aimed to identify any relevant studies, regardless of their quality.

### Data analysis

After coding and selecting the articles that would become eligible to review, we analyzed the data. All authors read the full texts of the preliminary articles and cross checked the validity and trustworthiness of the articles extracted. ERH and SY re-read the articles, manually highlighting and summarizing to capture meaningful aspects of the manuscripts. We highlighted the thesis in different colors according to the different trends in medical education, marking important content and main outcomes of the curriculum in the original text.

Then, we organized them through a two-step process. In the first step, we summarized the papers in terms of “trend,” “sub-trend,” “specific teaching and learning, curriculum, assessment, and technology,” “additional new trend or sub-trend (if any),” “additional journals to be sought from the reference list,” and “important reference for citation.” In the second step, three authors (ERH, SY, and HR) developed a comprehensive literature matrix, including significant information related to the author, country, study design, title of the program, learners, objectives of the program, duration of the program, location of education, learning resources, and outcomes of the program (Table 1).

**Table 1:** Description of the articles in the integrative review

Author (year)	Country	Study design	Title of program	Learners	Objectives of program	Duration of program	Location of education	Learning resources	Outcomes of program	Quality of study
Shield et al. (2011) [21]	USA	Descriptive study	Schwartz Communication Sessions	1st- and 2nd-year MS	To improve communication skills	2 years	School	Patients, families, and the health care team	Eagerness to learn practical ways to communicate with patients in real life	Good
Solomon et al. (2011) [22]	Canada	Descriptive study	Professional Competencies Course	1st- and 2nd-year MS	To raise awareness of inter-professional care in the home	2 terms (final term of 1st year and first term of 2nd year)	Patients’ homes	Clinical preceptors from different professions	A greater understanding of the patients’ perspective and determinants of health; an appreciation of the importance of collaboration	Good
de Boer et al. (2011) [23]	Netherlands	Experimental study	Real patient learning practicals	3rd-year MS	To demonstrate in practice the theory and to make students aware of the impact of a disease on patients’ lives	3 weeks	School	Real patients	Early contextualizing of the theory, better memorizing of clinical pictures, and deep understanding of the impact of the disease	Good
Pelling et al. (2011) [24]	Sweden	Descriptive study	Inter-professional training ward	MS and other health profession students	To train students to become proficient in teamwork	2 weeks	Orthopedic ward	Patients, clinical supervisors (nurse, PT, OT, orthopedic surgeon)	A higher level of insight into their own and other professional roles, and the importance of teamwork within health care	Fair
Schillerstrom et al. (2012) [25]	USA	Descriptive study	Death-and-Dying Human Behavior Course	1st-year MS	To improve students’ comfort with and knowledge of end-of-life issues	2 h of lectures and 2 h of small-group activities	School	Family members of recently deceased loved ones and faculty	Meaningful experience to decrease distress and improve end-of-life knowledge at an early point	Fair
Hirsh et al. (2012) [26]	USA	Experimental study	Harvard Medical School–Cambridge Integrated Clerkship	3rd-year MS	To learn the core skills of doctoring by following a panel of patients	1 year	Ambulatory care setting	Same patients and faculty	Richer perspectives on the course of illness, more insight into social determinants of illness and recovery, and increased commitment to patients	Good
Woodard et al. (2012) [27]	USA	Descriptive study	Primary Care and Special Populations clerkship	3rd-year MS	To be more competent and comfortable with people with disabilities	12 weeks	Community site, patient’s home, classroom	Patients with physical or intellectual disabilities	Improved knowledge, attitudes, and comfort in caring for people with disabilities	Fair
Teherani et al. (2013) [28]	USA	Experimental study	Longitudinal integrated clerkship	3rd-year MS	To participate in the care of patients over time and develop learning relationships with clinicians	1 year	Largely ambulatory setting	Same patients, peers, and faculty	Strengthening patient-centeredness and student-driven learning through continuity with patients, peers, and faculty	Good
Chastonay et al. (2013) [29]	Switzerland	Descriptive study	Community immersion clerkship	3rd-year MS	To be capable of working in and with the community and understand the communities their patients live in	4–6 weeks	Community	Community health workers, community health institutions, and patients’ families	Responding to the health problems of individuals in terms of their complexity and strengthening the ability to work with the community	Good
O’Neill et al. (2013) [30]	USA	Descriptive study	Education-Centered Medical Home (a longitudinal clerkship)	1st–4th-year MS	To introduce the concepts and process of QI under the supervision of a preceptor	1 year	Outpatient clinics	High-risk patients, preceptors	Participating in firsthand health care quality measurement and identifying opportunities to improve the quality of patient care	Good
Alamodi et al. (2014) [31]	Saudi Arabia	Descriptive study	Student-driven undergraduate research committee	MS	To promote, sustain and, improve undergraduate research environments	Whole academic year	On-campus, off-campus	International or local researcher	Capability to choose research areas of their own interest and develop basic skills in research conduct	Fair
Warde et al. (2014) [32]	USA	Descriptive study	Leadership course in UCLA PRIME Program	1st-year MS	To foster leadership, advocacy, and resiliency	3 weeks	Community	Medically underserved populations	Improved mindfulness and team relational coordination	Fair
Sheline et al. (2014) [33]	USA	Descriptive study	Primary Care Leadership Track	MS	To provide the knowledge, skills, and attitudes necessary to improve both health and future health care	4 years	Community	Faculty, community health professionals, and patients	Engaging with the community and exploring solutions to address the health of the public and the future delivery of health care	Fair
Potash et al. (2014) [34]	Hong Kong	Experimental study	Arts-making workshop in a family medicine clerkship	3rd-year MS	To develop empathic understanding of patients	3 h	Clinics	Faculty as well as a qualified art therapist	Fostering meaningful reflection and greater self-awareness	Good
Kalen et al. (2015) [35]	Sweden	Descriptive study	Longitudinal mentoring program	MS	To facilitate students’ professional and personal development	5.5 years	School, mentors’ clinic	Physician mentors, patients	Imaging their future life as a physician and learning about the physician’s doings at an early stage of their education	Good
Ferguson et al. (2015) [36]	USA	Descriptive study	QI and Patient Safety Scholarly Pathway	1st–3rd year MS	To develop interest in gaining exposure to QI and patient safety concepts	2 years with an optional third year	School	Faculty mentors, patients, and institutional, regional leaders	Identifying systems- and process-based errors, and practicing disclosing the error to the patient’s family	Fair
Swanberg et al. (2015) [37]	USA	Descriptive study	Diversity Dialogue	MS	To promote cultural competence and raise awareness of health care disparities	3–4 dialogues (1.5 h in length) per year	School	Multidisciplinary team of librarians, faculty, and staff	Understanding diverse perspectives from physicians, patients, and non-profit organizations, and raising awareness of health disparity issues	Fair
Chou et al. (2016) [38]	Taiwan	Experimental study	Inter-professional problem-based clinical ethics	4th-year MS, 3rd-year nursing students	To balance their socialized viewpoints by seeing ethical dilemmas from others’ standpoints	Two 2-h tutorial sessions	School	Problem-based clinical ethics	Recognizing different viewpoints from other professionals, and realizing the need to know each other and collaborate on delivering care to patients	Good
Milford et al. (2016) [39]	USA	Descriptive study	Collaboration with Head Start	1st- and 2nd-year MS	To improve students’ attitudes, knowledge, and skills in health literacy	2 h per week for 7 months (an academic year)	Community	Head Start population	Truly understanding the barriers created by poor health literacy and poverty, and effectively training in how to put the changed attitudes into action	Good
Chen et al. (2016) [40]	USA	Descriptive study	Medical student–Faculty collaborative clinics	Junior (1st or 2nd year), Senior MS (3rd or 4th year)	To engage in active experiential learning and systems-based practice training	6 months	Clinics	Patient Visit Tracker (software), patients, attending physicians	Being able to identify bottlenecks in the system, propose solutions, and then test the efficacy of their interventions	Fair
Pettignano et al. (2017) [41]	USA	Descriptive study	Interprofessional medical–legal education	3rd-year MS, law students	To identify social determinants of health with potential legal solutions	4 sessions (2 h in length per session)	School	Faculty, staff attorneys	Understanding the importance of identifying health-harming legal problems and of advocating for the inclusion of lawyers on care coordination teams	Fair
van der Meulen et al. (2017) [42]	Netherlands	Descriptive study	Gender health issues in the Nijmegen medical curriculum	1st–3rd-year MS	To learn about the effects of gender health issues in medical care	8 courses (2–4 weeks per course)	School	Integrated gender perspective in the medical curriculum	Being aware of gender differences in biomedical and social contexts, and understanding the role of their own gender in their profession as doctors	Fair
Mwenda (2012) [43]	Kenya, Sweden	Descriptive study	Moi–Linköping exchange programme	MS and other health professional students	To understand the differences in the health care system and enhance the global outlook to health	6 weeks in Kenya, 12 weeks in Sweden	Another country	Different health care system	Broadening students’ learning platform and exposing them to cultural and health care organization diversity	Fair
Johnson et al. (2013) [44]	USA	Experimental study	Virtual patient simulators	2nd-year MS	To practice diagnosis formulation of rare and complex medical conditions	–	Online community	Computer-based clinical scenarios	Facilitating student learning and engagement in team-based learning without risk of patient harm	Good
Kaltman et al. (2015) [45]	USA	Experimental study	Motivational Interviewing Training in a family medicine clerkship	3rd-year MS	To provide training in the widely dispersed student-preceptor placements	4 weeks	Clinics, online community	Online learning community	Enhancing students’ learning by providing a video recording of a live patient encounter and individualized feedback without a burden on faculty time	Good
O’Donovan et al. (2015) [46]	UK, Malaysia	Descriptive study	Distant peer-tutoring of clinical skills	2nd-year MS from Malaysia and 4th-year MS from UK	To facilitate peer-to-peer tutoring in clinical skills between students in two different countries	3 weeks	Online community	Distant peer tutor, online learning community	Encouraging active learning and building a strong rapport using a low-cost, time efficient, and easily accessible education tool in resource-limited settings	Good
Ma et al. (2016) [47]	Germany	Descriptive study	Magic mirror	MS	For anatomy education through personalized and interactive augmented reality	–	E-learning	Augmented reality	Facilitating autonomous and interactive learning by close-to-reality presentation without using laboratory materials and costs	Fair
Keynejad et al. (2016) [48]	UK, Somaliland	Descriptive study	Peer-to-peer e-learning	3rd-year UK, 3rd–5th-year Somaliland MS	To strengthen health care systems in low- and middle-income countries through mutual exchange	Ten times for 1 h	E-learning	E-learning, peer tutor	Creating low-cost opportunities for cross-cultural learning in restricted medical education and health care resources	Good

*MS* Medical students, *PT* Physiotherapist, *OT* Occupational therapist, *UCLA PRIME program* University of California at Los Angeles Program in Medical Education program, *QI* Quality improvement

We analyzed the extracted articles and compared them item by item to categorize and group them together. All authors were able to reach a consensus on the themes and subthemes to be generated.

## Results

We selected 28 articles that reflected medical education trends and covered suitable educational programs. Descriptions of the selected articles in this integrative review are shown in Table 1. Fifteen studies were conducted in the United States of America; three in Sweden; two each in the Netherlands and the United Kingdom; and one each in Canada, Switzerland, Saudi Arabia, Hong Kong, Taiwan, Kenya, Malaysia, Germany, and Somaliland (three of the studies were conducted in two countries). Twenty-one studies were descriptive and seven were experimental. The quality was evaluated as “good” in 15 studies and “fair” in 13 studies.

The integrative themes of future medical education are as follows: 1) humanistic approach to patient safety, 2) early experience and longitudinal integration, 3) beyond hospitals, toward society, and 4) student-driven learning with advanced technology (Table 2).

**Table 2:** Themes and subthemes of medical education trends for future physicians in the integrative review of the literature

Trend	Reference number
Humanistic approach to patient safety	
Encouraging humanistic doctors	[21–26, 28, 29, 32–35, 39]
Facilitating collaboration	[22, 24, 25, 27–29, 32, 36–38, 41]
Early experience and longitudinal integration	
Early exposure to patient-oriented integration	[21–25, 30, 31, 35, 36, 39, 40]
Longitudinal integrated clerkships	[26–28, 30, 33]
Beyond hospitals, toward society	
Responding to changing community needs	[22, 26, 27, 29, 32, 33, 39, 41, 48]
Respect for diversity	[26, 27, 37, 39, 42, 43, 48]
Student-driven learning with advanced technology	
Active learning with individualization	[44–47]
Social interaction	[44–46, 48]
Resource accessibility	[43, 45, 46, 48]

### Humanistic approach to patient safety

First of all, many medical educators have taken a humanistic approach to helping future physicians learn to interact with patients and collaborate with health professionals in their clinical practice to ensure patient safety.

#### Encouraging humanistic doctors

Students have been encouraged to develop into humanistic doctors who can have a better understanding of patients, have a deeper level of learning about physicians’ doings, and build meaningful relationships with patients in real or realistic clinical settings [21–26, 28, 29, 32–35, 39]. The five detailed educational programs to encourage humanistic doctors are as below. Communication sessions within the pre-clerkship course provided students with real clinical case discussions, composed of patients, families, and an expert panel consisting of a physician, social worker, and chaplain. This helped students appreciate and understand the medical and ethical complexities of the patient, and communicate better with patients and family members [21]. Death-and-dying discussions with family members of the recently deceased and with mental-health and palliative-medicine faculty members were introduced to first-year students and led to the acquisition of comfort with and knowledge of end-of-life issues at the early stage of their careers [25]. An underserved community-based service project coupled with a relationship-centered leadership course promoted students’ mindfulness and coordination as characteristics of effective and resilient leaders [32]. In an arts-making workshop during the clerkship, students wrote a poem, created artwork based on the poem, and completed a reflective essay to describe a memory of a patient in pain or suffering. This helped students develop empathic understanding of patients and increased their emotional awareness [34]. Longitudinal mentorship offered opportunities for students to visit the mentor in connection with their clinical work and helped them imagine their future life as a physician and learn how to interact with patients with complex psychosocial needs and prepare for that in the process of becoming a physician [35].

#### Facilitating collaboration

Hands-on experience of inter-professional collaboration enabled medical students to appreciate their professional roles, respect others’ standpoints, and be aware of the necessity of collaborating with other health professionals for patient safety [22, 24, 25, 27–29, 32, 36–38, 41]. The three representative examples of facilitating collaboration are as follows. An orthopedic inter-professional training ward offered the medical students a chance to practice with a team consisting of nursing, physiotherapy, and occupational therapy students, and establish a holistic view of the patients and strengthen their insight into their own and other professional roles [24]. Inter-professional problem-based clinical ethics allowed medical students to experience clinical ethical dilemmas with nursing students and respect different viewpoints [38]. Through inter-professional learning in home care settings, preclinical students visited patient homes with preceptors from different professions including occupational therapists, physiotherapists, registered dieticians, speech language pathologists, and social workers. They gained an appreciation of how important it is to coordinate resources as a team to provide the best care, in addition to a deeper understanding of the patient’s perspective [22].

The challenges of humanistic approach to patient safety would be to develop reliable and valid methods to assess students’ outcomes, which are relevant to the educational programs in the short term and long term [22, 24, 25, 32, 34, 38]. Besides, medical educators should consider how to persuade the faculty and students to participate in these programs, particularly those who have false beliefs that humanistic aspects are innate and immutable [21].

### Early experience and longitudinal integration

Early experience of patient contact and longitudinal integration of clinical practice have been promoted as ways to improve students’ attitudes toward patients and the consequent quality of patient care, as well as students’ motivation and learning.

#### Early exposure to patient-oriented integration

Early integration of a theory into clinical reality would be an initial step forward in the improvement of quality of patient care [21–25, 30, 31, 35, 36, 39, 40]. The early integrated programs were as follows. Real patient learning practicals, which were integrated into the preclinical block lectures, enabled students to examine real patients earlier, memorize the clinical pictures by contextualizing the theory, and understand the impact of disease [23]. The Quality Improvement (QI) and Patient Safety Scholarly Pathway was longitudinally incorporated into the existing curriculum, which was a faculty-mentored, three-year experience for students to identify systems- and process-based errors and practice then disclose the error to the patient’s family [36]. Medical Student–Faculty Collaborative Clinics allowed students to engage in systems-based practice, design care processes first-hand, and create solutions for inefficiencies in clinic operations through a patient visit tracker tool [40]. A student-driven undergraduate research committee offered students opportunities to acquire basic research skills through theoretical and practical sessions, and provided a crucial platform for research training to the new generation of physician-scientists [31].

#### Longitudinal integrated clerkships

Longitudinal integrated clerkships (LIC) enabled students to foster practice-based learning, reinforce patient-centeredness, and perceive safe multidisciplinary care by continuously following a panel of patients with the same faculty and peers at a single site [26–28, 30, 33]. LIC helped students develop richer perspectives on the course of illness and more insight into social determinants of illness and recovery, and establish meaningful relationships with their patients [26]. Students preferred to see patients and work with a stable group of peers and faculty mentors within a community over time; this contributed to their learning and understanding of patient care and the health care system [28, 33]. An authentic QI curriculum embedded in a longitudinal clerkship allowed the students to see their clinic’s performance outcomes and identify opportunities to improve the quality of care delivered to the patients [30].

In terms of early experience and longitudinal integration, medical educators should consider the expansion of the relevant programs to all students and incorporating them into the regular curriculum [23, 31, 36, 40]. They also should investigate whether the effect of programs would be sustained [26, 30].

### Beyond hospitals, toward society

Beyond hospitals, medical students have been encouraged to go out into society to meet the specific needs of the community and interact with diverse patients.

#### Responding to changing community needs

Changing communities require future physicians to respond to their needs appropriately [22, 26, 27, 29, 32, 33, 39, 41, 48]. Educational programs in community settings are as follows. The community immersion clerkship allowed preclinical medical students to investigate complex health problems from biopsychosocial perspectives by getting directly in touch with, interviewing, and interacting with various community health institutions or community actors (politicians, opinion leaders, associations, and nongovernmental organizations), as well as meeting with concerned patients and families [29]. The community immersion project was also an effective way for preclinical students to begin to truly understand the barriers created by poor health literacy and poverty, and to train future physicians in how to put these changed attitudes into action [39]. Inter-professional medical legal education between medical and law students can improve medical students’ ability to identify and address medical, social, and even legal issues affecting health, and to advocate for the inclusion of lawyers in care coordination teams to help improve the health status of their patients [41].

#### Respect for diversity

Respect for diversity should be obtained in order to understand the specific health demands of diverse patients and make better decisions for them [26, 27, 37, 39, 42, 43, 48]. Here are three examples of openness to diversity. The diversity dialogue program allowed medical students to participate in a multidisciplinary team consisting of librarians, faculty and staff, and invited patients. Here, they could hear different perspectives and raise their awareness of health care disparities [37]. The longitudinal primary care and special populations clerkship allowed for students to interact with elders and patients with physical or intellectual disabilities, dispel commonly held negative assumptions about their quality of life, and learn to be sensitive to the needs created by the disability [27]. Gender issues have been constantly present in undergraduate medical education; students are taught to be aware of gender differences in biomedical and social contexts, and to understand the role of their own gender in their profession as doctors [42].

Medical educators would be faced with the same challenges to develop reliable and valid methods to assess students’ outcomes that are relevant to the educational programs in the long term [37, 39, 41] and maintain the programs to incorporate them into the regular curriculum [27, 37, 42].

### Student-driven learning with advanced technology

Advanced technology facilitates students’ learning by providing learning opportunities whenever they want to learn more to meet their own needs, to whoever wants to interact with their peers and faculty to share valuable information, and with whatever resources they can access regardless of geographic location.

#### Active learning with individualization

High technology has allowed individualized learning by increasing students’ interest [44–47]. Virtual patient simulators facilitate students’ learning as they offer venues for practicing the diagnosis of medical abnormalities without risk of patient harm and for observing abnormal pathology not otherwise readily available through live patient encounters [44]. Personalized augmented reality systems could also help to promote autonomous learning by reducing laboratory materials and instructor costs [47]. These resources are more interactive and interesting than textbooks because information can be embedded and/or superimposed upon reality [44, 47].

#### Social interaction

Technology has also facilitated social interaction with peers and faculty through computers or mobile devices, anytime and anywhere [44–46, 48]. One online learning community provided students with an opportunity to practice clinical skills and interact with peers and faculty via student-initiated video recordings of a live patient encounter using readily available devices and individualized feedback from faculty, in spite of widely dispersed student-faculty placements and the heavy schedules of faculty during the clerkship [45]. Mobile devices used to deliver video tutorials and remote online peer-tutoring in clinical skills enabled the students to build a good rapport and enjoy distance learning between two continents at a time convenient to both tutors and learners [46].

#### Resource accessibility

Advanced technology has enabled students in resource-limited settings to connect to other learners, faculty, and even other curricula [43, 45, 46, 48]. Distant peer-to-peer e-learning has encouraged students to expand the scope of health beyond the limit of their resources and to understand how heath care works differently in different cultures [48]. An exchange program offered students the chance to experience the organization and delivery of health care in the country of exchange and be aware of significant cultural and health care organization diversity [43].

The last approach would be confronted with technical problems such as errors in computer programs and unstable Internet connection as a lack of infrastructure in developing countries, and difficulties to find mutually suitable time for real-time communication because of a time lag in distant learning [43, 46, 48].

## Discussion

This review sought to synthesize the values that global medical educators are pursuing to foster future physicians, and to introduce feasible and concrete educational programs to enable students to become competent physicians. Our results emphasized the trends in future medical education in comparison to previous reports: leading medical students to become more humanistic and collaborate with others for patient safety, providing patient-oriented integration earlier and longitudinally, encouraging them to respond to the changing community needs and respect the diversity, and facilitating student-driven learning with the aid of advanced technology.

**Fig. 2:**
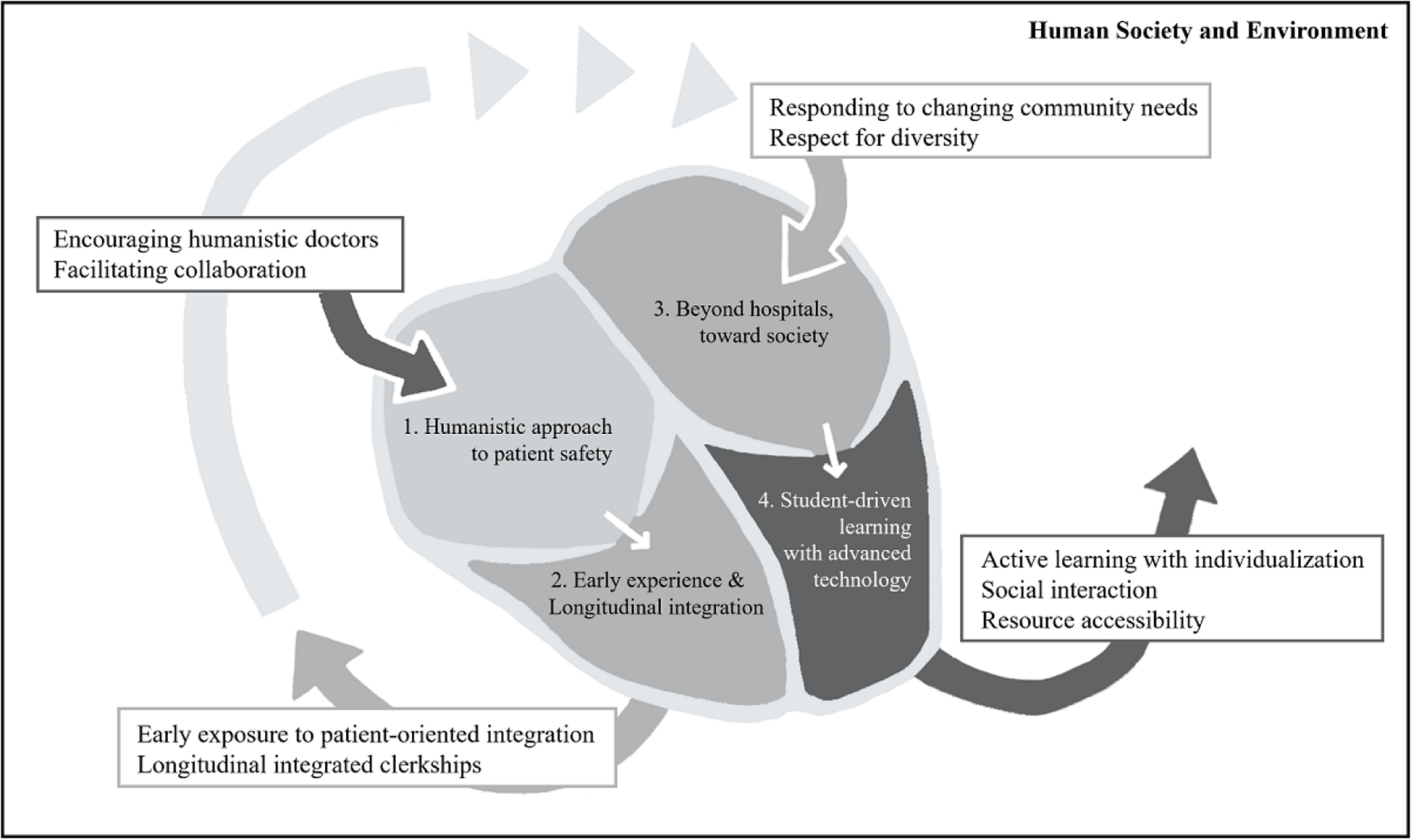
Trends at the heart of medical education for future physicians in advanced technology and artificial intelligence. As the heart serves as a pump to supply blood to our bodies, medical schools are the heart of medical education and provide competent physicians to our communities. When potential students are admitted to a medical school, the school cultivates in them a humanistic approach to patient safety. To encourage humanistic doctors and facilitate their collaboration with other professionals, students are exposed to early clinical experiences and the longitudinal integration of medical education in the school curricula. Beyond the general hospital affiliated to the school, the students inhale fresh air in the society so that they are well equipped to respond to the needs of changing communities and respect diverse patients. After students are developed in the powerful ventricle, which is a school armed with advanced technology, they can take a leap into the future society

In Fig. 2, we liken the four themes to blood flowing through the heart. As the heart serves as a pump to supply blood to our bodies, medical schools are the heart of medical education and provide competent physicians to our communities. When potential students are admitted to a medical school, the school cultivates in them a humanistic approach to patient safety. To encourage humanistic doctors and facilitate their collaboration with other professionals, students are exposed to early clinical experiences and the longitudinal integration of medical education in the school curricula. Beyond the general hospital affiliated to the school, the students inhale fresh air in the society so that they are well equipped to respond to the needs of changing communities and respect diverse patients. After students are developed in the powerful ventricle, which is a school armed with advanced technology, they can take a leap into the future society.

The most searched-for value in this review was a humanistic approach. Although times have changed, medical educators have and will continue to concentrate on students’ humanistic approach: interacting with patients compassionately and collaborating with health care teams. A humanistic approach can influence patients’ satisfaction, consolidate patients’ trust in their doctors, and improve health outcomes [49–53]. To learn and practice what they should do, the students were provided with encounters with real patients and their families or co-work with health professionals in clinical settings [21–29, 32–39, 41].

Second, we found that early experiences and longitudinal integration are important factors for the future. As technology is rapidly changing, medical education is moving toward integration in order to facilitate contextual and applied learning, and to develop problem-solving skills on the basis of uncertainty [54–56]. Early experiences and longitudinal integration helped students increase their motivation and deepen their understanding of patient care [23, 30, 31, 36, 40]. LIC facilitated students’ learning in regard to patient care, supervision, and curricula and eventually helped them enhance their patient-centered attitudes by following the patients longitudinally [26, 28, 30, 33].

Third, the learning environment in medical education has extended into society, beyond the confines of the teaching hospital. Future physicians will be faced with more complex health problems in society and more heterogeneous patient populations [29, 57, 58]. A community immersion program gave students the opportunity to get involved with community health institutions prior to the beginning of clinical clerkships [29]. Ultimately, hands-on experience of collaborating with the community enabled students to respond to a health problem with its biopsychosocial and cultural complexity and develop their social accountability and responsibility [29, 39, 41]. Incorporating health and social issues based on differences in gender, race, ethnicity, age, religion, and socioeconomic background into medical education has been highlighted as helping students learn to respect patient diversity and raising their awareness of health care disparities [27, 37, 42].

Last, learning materials equipped with advanced technology have been provided to students to drive individualized learning, interaction with peers and tutors, and access to rich sources of information. Virtual patient and augmented reality simulations can offer realistic medical conditions without risk of patient harm and facilitate students’ learning and engagement [44, 47]. Mobile and online learning are able to supplement students’ learning and enhance peer-to-peer or student-to-faculty interaction using readily available devices [45, 46]. Even in limited resource settings, e-learning helps students to connect with their peers, tutors, and curricula all over the continent [46, 48]. There are some considerations of digital learning with advanced technology, although it has many technical advantages. We must give careful consideration to ethical and moral challenges because computer-based learning and artificial intelligence algorithms may be programmed to be biased against certain groups or skewed toward any interests [59, 60]. Most of all, a humanistic approach should be prioritized for future physicians to deal with biopsychosocial complexity of patients that are not easily accessible to machines [60]. Especially in the distance learning, it is necessary to arrange mutually convenient times for interaction [46, 48]. Even if learners are in different geographical areas from instructors and other learners, online collaborative learning is effective when they have the feelings of connectedness with and belonging to others, that is, emotional bonding and support [61]. Nevertheless, it does not simply imply turning the traditional lectures to online collaborative learning because students’ motivation and their interaction depend on the course structure, which should be designed to encourage students engage in discussions and collaborate on projects [61].

We will now discuss the implications of these themes for medical education. First, we recommend that medical educators evaluate whether their curricula reflect these four values. Second, we suggest that educators find a creative way to apply and customize the examples of the educational programs discussed in their own educational environments across different educational systems and medical environments. Last but not least, we emphasize that it is important to start with a pilot program to see what they can implement.

This study had some limitations when it came to collecting eligible articles. Since this review extracted only published research, it might have missed educational interventions that medical educators are already implementing but have not yet published in the literature. For example, innovative methods that are not well-developed, such as the application of artificial intelligence in medical education, may not be published and hence not included in this study. Although we searched for articles using general key words, these were limited and relevant articles may have been excluded. Also, any articles written in a language other than English were excluded. However, most of the authors were found to have conceived of similar ideas on how to foster humanistic physicians when they encounter patients and other health professionals, what educational programs will be effective in integrating scientific knowledge into clinical practice, how to incorporate educational programs into society, and which advanced technology programs will be useful to drive students’ learning.

Further research is needed in several ways. First, research should investigate how to integrate educational trends into graduate and continuing medical education by extending and connecting these future trends in undergraduate medical education. Second, an observational study will be required to evaluate whether these educational values are incorporated in their own curricula. An experimental study will be necessary to investigate the effect of new innovative programs that are focused on these values and are customized for their own educational environment. Third, further studies should investigate in-depth digital learning in medical education and develop a critical digital literacy curriculum considering the relevant humanistic values and ethical standards because they are rapidly changing under the influence of digitalization.

## Conclusion

The pursuit of future medical education is about strengthening the humanistic approach to patients and other professional teams to ensure patient safety. Early clinical experience and longitudinal integration are very helpful in promoting effective and lifelong learning. Community-based programs enable students to broaden their perspectives on society and develop respect for diverse patients. Future physicians will be able to use high technology for individualized learning, social interaction, and access to vast resources.

This review integrated the educational trends in undergraduate medical education in preparation for the anticipated changes in medical environments. Details on the programs introduced in this study can be used by medical educators in the development of curricula. Medical educators would be challenged to develop reliable and valid methods to assess students’ outcomes, which are pertinent to the educational programs in the short and long term, and consider the expansion of the relevant programs to all students and incorporation of them into the regular curriculum.

## Data Availability

Not applicable.

## Abbreviations

LIC: Longitudinal integrated clerkships
MS: Medical students
OT: Occupational therapist
PBL: Problem-based learning
PT: Physiotherapist
QI: Quality Improvement
UCLA PRIME program: University of California at Los Angeles Program in Medical Education program

## References

[1] Friedman CP, Donaldson KM, Vantsevich AV (2016). Educating medical students in the era of ubiquitous information. Med Teach..

[2] Johnston SC (2018). Anticipating and training the physician of the future: the importance of caring in an age of artificial intelligence. Acad Med.

[3] Obermeyer Z, Emanuel EJ (2016). Predicting the future—big data, machine learning, and clinical medicine. N Engl J Med.

[4] Pershing S, Fuchs VR (2013). Restructuring medical education to meet current and future health care needs. Acad Med.

[5] Gushulak BD, Weekers J, MacPherson DW (2009). Migrants and emerging public health issues in a globalized world: threats, risks and challenges, an evidence-based framework. Emerg Health Threats J.

[6] Labonté R, Mohindra K, Schrecker T (2011). The growing impact of globalization for health and public health practice. Annu Rev Public Health.

[7] Bullen M, Morgan T. Digital learners not digital natives. La Cuestión Universitaria. 2011;7:60–8.

[8] Sandars J, Morrison C (2007). What is the net generation? The challenge for future medical education. Med Teach..

[9] Boysen PG, Daste L, Northern T (2016). Multigenerational challenges and the future of graduate medical education. Ochsner J.

[10] Borges NJ, Manuel RS, Elam CL, Jones BJ (2010). Differences in motives between millennial and generation X medical students. Med Educ.

[11] Greiner AC, Knebel E, Greiner AC, Knebel E (2003). Health Professions Education: A Bridge to Quality.

[12] Training Tomorrow’s Doctors: The Medical Education Mission of Academic Health Centers. a Report of the Commonwealth Fund Task Force on Academic Health Centers. New York: Commonwealth Fund; 2002.

[13] Irby DM, Cooke M, O'brien BC (2010). Calls for reform of medical education by the Carnegie Foundation for the Advancement of Teaching: 1910 and 2010. Acad Med.

[14] Hoy WK, Miskel CG (2013). Educational administration: theory, research, and practice.

[15] Whittemore R, Knafl K (2005). The integrative review: updated methodology. J Adv Nurs.

[16] Whittemore R, Chao A, Jang M, Minges KE, Park C (2014). Methods for knowledge synthesis: an overview. Heart Lung.

[17] Murdoch TB, Detsky AS (2013). The inevitable application of big data to health care. JAMA..

[18] Chen X-W, Lin X (2014). Big data deep learning: challenges and perspectives. IEEE access.

[19] Conn VS, Sa I, Rath S, Jantarakupt P, Wadhawan R, Dash Y (2003). Beyond medline for literature searches. J Nurs Scholarsh.

[20] Hawker S, Payne S, Kerr C, Hardey M, Powell J (2002). Appraising the evidence: reviewing disparate data systematically. Qual Health Res.

[21] Shield RR, Tong I, Tomas M, Besdine RW (2011). Teaching communication and compassionate care skills: an innovative curriculum for pre-clerkship medical students. Med Teach..

[22] Solomon P, Risdon C (2011). Promoting interprofessional learning with medical students in home care settings. Med Teach.

[23] de Boer A, Melchers D, Vink S, Dekker F, Beaart L, de Jong Z (2011). Real patient learning integrated in a preclinical block musculoskeletal disorders. Does it make a difference?. Clin Rheumatol.

[24] Pelling S, Kalen A, Hammar M, Wahlstrom O (2011). Preparation for becoming members of health care teams: findings from a 5-year evaluation of a student interprofessional training ward. J Interprof Care.

[25] Schillerstrom JE, Sanchez-Reilly S, O'Donnell L (2012). Improving student comfort with death and dying discussions through facilitated family encounters. Acad Psychiatry.

[26] Hirsh D, Gaufberg E, Ogur B, Cohen P, Krupat E, Cox M (2012). Educational outcomes of the Harvard Medical School-Cambridge integrated clerkship: a way forward for medical education. Acad Med.

[27] Woodard LJ, Havercamp SM, Zwygart KK, Perkins EA (2012). An innovative clerkship module focused on patients with disabilities. Acad Med.

[28] Teherani A, Irby DM, Loeser H (2013). Outcomes of different clerkship models: longitudinal integrated, hybrid, and Block. Acad Med.

[29] Chastonay P, Zesiger V, Klohn A, Soguel L, Mpinga EK, Vu N (2013). Development and evaluation of a community immersion program during preclinical medical studies: a 15-year experience at the University of Geneva Medical School. Adv Med Educ Pract.

[30] O'Neill SM, Henschen BL, Unger ED, Jansson PS, Unti K, Bortoletto P (2013). Educating future physicians to track health care quality: feasibility and perceived impact of a health care quality report card for medical students. Acad Med.

[31] Alamodi AA, Abu-Zaid A, Anwer LA, Khan TA, Shareef MA, Shamia AA (2014). Undergraduate research: an innovative student-centered committee from the Kingdom of Saudi Arabia. Med Teach..

[32] Warde CM, Vermillion M, Uijtdehaage S (2014). A medical student leadership course led to teamwork, advocacy, and mindfulness. Fam Med.

[33] Sheline B, Tran AN, Jackson J, Peyser B, Rogers S, Engle D (2014). The primary care leadership track at the Duke University School of Medicine: creating change agents to improve population health. Acad Med.

[34] Potash JS, Chen JY, Lam CL, Chau VT (2014). Art-making in a family medicine clerkship: how does it affect medical student empathy?. BMC Med Educ.

[35] Kalen S, Ponzer S, Seeberger A, Kiessling A, Silen C (2015). Longitudinal mentorship to support the development of medical students’ future professional role: a qualitative study. BMC Med Educ..

[36] Ferguson CC, Lamb G (2015). A scholarly pathway in quality improvement and patient safety. Acad Med.

[37] Swanberg SM, Abuelroos D, Dabaja E, Jurva S, Martin K, McCarron J (2015). Partnership for Diversity: a multidisciplinary approach to nurturing cultural competence at an emerging medical school. Med Ref Serv Q.

[38] Chou FC, Kwan CY, Hsin DH (2016). Examining the effects of interprofessional problem-based clinical ethics: findings from a mixed methods study. J Interprof Care..

[39] Milford E, Morrison K, Teutsch C, Nelson BB, Herman A, King M (2016). Out of the classroom and into the community: medical students consolidate learning about health literacy through collaboration with head start. BMC Med Educ..

[40] Chen CA, Park RJ, Hegde JV, Jun T, Christman MP, Yoo SM (2016). How we used a patient visit tracker tool to advance experiential learning in systems-based practice and quality improvement in a medical student clinic. Med Teach..

[41] Pettignano R, Bliss L, McLaren S, Caley S (2017). Interprofessional medical-legal education of medical students: assessing the benefits for addressing social determinants of health. Acad Med.

[42] van der Meulen F, Fluit C, Albers M, Laan R, Lagro-Janssen A (2017). Successfully sustaining sex and gender issues in undergraduate medical education: a case study. Adv Health Sci Educ Theory Pract.

[43] Mwenda A (2012). A Student’s Analysis of the Moi University-Linköping University Exchange Programme. Educ Health (Abingdon).

[44] Johnson TR, Lyons R, Chuah JH, Kopper R, Lok BC, Cendan JC (2013). Optimal learning in a virtual patient simulation of cranial nerve palsies: the interaction between social learning context and student aptitude. Med Teach..

[45] Kaltman S, WinklerPrins V, Serrano A, Talisman N (2015). Enhancing motivational interviewing training in a family medicine clerkship. Teach Learn Med.

[46] O’Donovan J, Maruthappu M (2015). Distant peer-tutoring of clinical skills, using tablets with instructional videos and Skype: a pilot study in the UK and Malaysia. Med Teach..

[47] Ma M, Fallavollita P, Seelbach I, Von Der Heide AM, Euler E, Waschke J (2016). Personalized augmented reality for anatomy education. Clin Anat.

[48] Keynejad R, Garratt E, Adem G, Finlayson A, Whitwell S, Sheriff RS (2016). Improved attitudes to psychiatry: a global mental health peer-to-peer E-learning partnership. Acad Psychiatry.

[49] Henman M, Butow P, Boyle F, Tattersall M (2002). Lay constructions of decision-making in cancer. Psychooncology..

[50] Rost K (1988). The influence of patient participation on satisfaction and compliance. Diabetes Educ.

[51] Kaplan SH, Greenfield S, Ware JE (1989). Assessing the effects of physician-patient interactions on the outcomes of chronic disease. Med Care.

[52] Del Canale S, Louis DZ, Maio V, Wang X, Rossi G, Hojat M (2012). The relationship between physician empathy and disease complications: an empirical study of primary care physicians and their diabetic patients in Parma, Italy. Acad Med.

[53] Farin E, Gramm L, Schmidt E (2013). The patient–physician relationship in patients with chronic low back pain as a predictor of outcomes after rehabilitation. J Behav Med.

[54] Harden RM, Sowden S, Dunn WR (1984). Educational strategies in curriculum development: the SPICES model. Med Educ.

[55] Moore GT, Block SD, Style CB, Mitchell R (1994). The influence of the new pathway curriculum on Harvard medical students. Acad Med.

[56] Barrows HS, Tamblyn RM. Problem-based learning: an approach to medical education: Springer Publishing Company; 1980.

[57] Liaison Committee on Medical Education. Functions and structure of a medical school: Standards for accreditation of medical education programs leading to the MD Degree. Washington, DC: Liaison Committee on Medical Education; 2016.

[58] Kumagai AK, Lypson ML (2009). Beyond cultural competence: critical consciousness, social justice, and multicultural education. Acad Med.

[59] Char DS, Shah NH, Magnus D (2018). Implementing machine learning in health care—addressing ethical challenges. N Engl J Med.

[60] Goldhahn J, Rampton V, Spinas GA (2018). Could artificial intelligence make doctors obsolete?. BMJ..

[61] So H-J, Brush TA (2008). Student perceptions of collaborative learning, social presence and satisfaction in a blended learning environment: relationships and critical factors. Comput Educ.

